# Non-invasive measurements of exhaled NO and CO associated with methacholine responses in mice

**DOI:** 10.1186/1465-9921-9-45

**Published:** 2008-05-27

**Authors:** Jigme M Sethi, Augustine MK Choi, William J Calhoun, Bill T Ameredes

**Affiliations:** 1 Division of Pulmonary, Allergy, and Critical Care Medicine, University of Pittsburgh School of Medicine, Pittsburgh, PA 15213, USA; 2 Division of Allergy, Pulmonary, Immunology, Critical Care and Sleep (APICS) Division, Department of Internal Medicine, University of Texas Medical Branch, Galveston TX 77755-1083, USA

## Abstract

**Background:**

Nitric oxide (NO) and carbon monoxide (CO) in exhaled breath are considered obtainable biomarkers of physiologic mechanisms. Therefore, obtaining their measures simply, non-invasively, and repeatedly, is of interest, and was the purpose of the current study.

**Methods:**

Expired NO (E_NO_) and CO (E_CO_) were measured non-invasively using a gas micro-analyzer on several strains of mice (C57Bl6, IL-10^-/-^, A/J, MKK3^-/-^, JNK1^-/-^, NOS-2^-/- ^and NOS-3^-/-^) with and without allergic airway inflammation (AI) induced by ovalbumin systemic sensitization and aerosol challenge, compared using independent-sample t-tests between groups, and repeated measures analysis of variance (ANOVA) within groups over time of inflammation induction. E_NO _and E_CO _were also measured in C57Bl6 and IL-10-/- mice, ages 8–58 weeks old, the relationship of which was determined by regression analysis. S-methionyl-L-thiocitrulline (SMTC), and tin protoporphyrin (SnPP) were used to inhibit neuronal/constitutive NOS-1 and heme-oxygenase, respectively, and alter NO and CO production, respectively, as assessed by paired t-tests. Methacholine-associated airway responses (AR) were measured by the enhanced pause method, with comparisons by repeated measures ANOVA and post-hoc testing.

**Results:**

E_NO _was significantly elevated in naïve IL-10^-/- ^(9–14 ppb) and NOS-2^-/- ^(16 ppb) mice as compared to others (average: 5–8 ppb), whereas E_CO _was significantly higher in naïve A/J, NOS-3^-/- ^(3–4 ppm), and MKK3^-/- ^(4–5 ppm) mice, as compared to others (average: 2.5 ppm). As compared to C57Bl6 mice, AR of IL-10^-/-^, JNK1^-/-^, NOS-2^-/-^, and NOS-3^-/- ^mice were decreased, whereas they were greater for A/J and MKK3^-/- ^mice. SMTC significantly decreased E_NO _by ~30%, but did not change AR in NOS-2^-/- ^mice. SnPP reduced E_CO _in C57Bl6 and IL-10^-/- ^mice, and increased AR in NOS-2^-/- ^mice. E_NO _decreased as a function of age in IL-10^-/- ^mice, remaining unchanged in C57Bl6 mice.

**Conclusion:**

These results are consistent with the ideas that: 1) E_NO _is associated with mouse strain and knockout differences in NO production and AR, 2) alterations of E_NO _and E_CO _can be measured non-invasively with induction of allergic AI or inhibition of key gas-producing enzymes, and 3) alterations in AR may be dependent on the relative balance of NO and CO in the airway.

## Background

Nitric oxide (NO) and carbon monoxide (CO) are small gaseous molecules produced physiologically in minute quantities, and are known to have significant physiologic effects, such as vasodilation and bronchodilation [1-5]. NO is produced by nitric oxide synthase (NOS), an enzyme present in numerous cells; the levels and activity of one inducible isoform (NOS-2) can be significantly modulated upward, by stresses such as inflammation [6,7]. Similarly, CO is produced by heme-oxygenase (HO), a ubiquitous enzyme within the body, and like NOS-2, HO-1 is an isoform of HO that can be upregulated by cellular stresses [8].

Recent technologies have allowed measurement of NO and CO within a variety of gaseous and liquid media, which has sparked interest in non-invasive assessments of their levels in expired gas. The findings that humans with asthma have elevated exhaled NO (E_NO_) which rises further with antigen challenge and asthmatic exacerbations [9], and which declines with anti-inflammatory therapy such as inhaled steroids [10], has established an association between E_NO _and airway inflammation, although the precise nature of the association remains unclear Similarly, increased exhaled CO (E_CO_) is associated with asthma and airway inflammation [11], and can be decreased with inhaled and oral steroids [12,13], although the association between E_CO _and asthma is less clear than that of E_NO _[14,15], and may be dependent on asthma severity [16]. In furthering the understanding of the relationship of E_NO _and E_CO _to lung function and airway inflammation, studies in which certain inflammation-modulating factors are lacking or suppressed, are of interest. Mice with genetically targeted deletions of these factors provide an opportunity to study their role in the determination of E_NO _and E_CO_, in association with airway inflammation and responsiveness.

Some prior studies have evaluated E_NO _and E_CO _in mice and rats using various techniques of expired gas collection, and including more invasive approaches, such as sampling collected gas from tracheostomy tubes during mechanical ventilation [17], and non-invasive approaches such as sampling expired gas within a flow-through chamber setup [18], with mild to moderate restraint [19]. Simple, non-invasive approaches have considerable appeal, because they allow repetitive sampling of unanesthetized individuals, e.g., with the development of inflammation and the potential alteration of lung function with experimental manipulations

Therefore, the purpose of the present study was to assess E_NO _and E_CO _using a simplified, non-invasive technique in spontaneously-breathing unanesthetized mice, in association with non-invasive assessments of airway responses (AR). Stock C57Bl6 and A/J mouse strains were studied, as well as those having targeted deletion of a critical inflammation-associated interleukin (IL)-10, cellular enzymes thought to be important in airway function (NOS-2 and NOS-3), and protein kinases mitogen-activated kinase kinase (MKK)-3, and c-jun activated kinase (JNK)1 which are known to mediate aspects of allergic AI and AR. The hypothesis was that non-invasively assessed airway NO and CO production are attributable to mechanisms associated with the above factors, and would be measurable as altered E_NO _and E_CO _levels as a function of those genetic manipulations. The results indicate that simple, non-invasively-measured levels of E_NO _and E_CO _can be associated with mouse genetic strain differences and non-invasively-measured AR, as well as aging and allergic AI responses.

## Methods

### General

Male mice of the C57Bl6 and A/J strains, and certified double knockout IL-10 null mutants (IL-10^-/-^) mice (B6.129P2-IL-10<tmlCgn> on a C57Bl6 background) [20] (Jackson Laboratories) were housed in a specific pathogen-free (SPF)-barrier facility in the Division of Laboratory Animal Resources Central Animal Facility, at the University of Pittsburgh. Mice with specific targeted disruption of the inducible NOS (NOS-2) gene (iNOS^-/-^) and the endothelial NOS (NOS-3) gene (eNOS^-/-^) [21] on a C57Bl6 background were obtained from breeding colonies grown under specific pathogen free (SPF) conditions at the University of Pittsburgh, and housed as described above. Mice with targeted deletion of MAP kinase kinase-3 (MKK3^-/-^) and c-Jun NH_2_-terminal kinase 1 (JNK1^-/-^) were obtained from breeders originating at Yale University, subsequently bred and grown under SPF conditions at the University of Pittsburgh, housed as described above. Littermate MKK3-sufficient mice (C57/C129) were obtained from within these same breeding colonies and housed as above. The A/J mouse strain was utilized for contrast with the C57Bl6 wild type and targeted gene knockout mice, as a strain known for its airway hyperresponsiveness [22]. All mice were allowed to age to 8–10 weeks before being subjected to the protocols described below, allowing them to grow to a size (18–25 grams) that facilitated airway cell recovery by bronchoalveolar lavage (BAL). All mice had constant access to Purina mouse chow and water, ad libitum. All procedures and protocols utilized in these studies were approved by the University of Pittsburgh Institutional Animal Care and Use Committee, conforming to guidelines recommended by the National Institutes of Health and the United States Department of Agriculture.

### Exhaled Nitric Oxide and Carbon Monoxide

**Figure 1:**
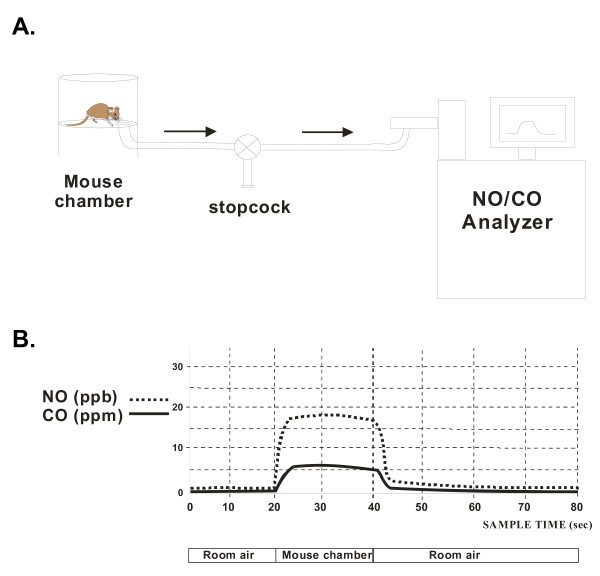
Schematics of exhaled gas setup and data. **A**. Schematic of experimental setup used to measure exhaled NO and CO in mice. Expired gas from the mouse was sampled from small holding chamber, with an integral stopcock to allow rapid sample switching from ambient to expired air. **B**. Schematic representation of signals redrawn from actual signals of one individual mouse with airway inflammation. Increased NO (16–18 ppb) and CO (5–7 ppm) is apparent in first 10s of 20s sampling period, when sampling chamber air, as compared with low concentrations of NO (0–2 ppb) and CO (0–1 ppm) in ambient air, pre- and post-sampling of chamber. Additional details are in text.

E_NO _and E_CO _were assessed by a multi-channel gas analyzer (Logan 2500, Logan Research Limited, Kent, UK), which measured E_CO _(sensitivity = 0.1 ppm, T_90 _<5 s), and E_NO _(sensitivity = 0.3 ppb, T_90 _<0.5 s), using photometric determination of chemiluminiscence. The same instrument measured CO_2 _concentrations with a sensitivity of 0.1%, and a T_90 _of 200 ms, using the infrared absorption principle. Unanesthetized mice were placed individually within a closed Plexiglas chamber with a low volume (~600 ml; Buxco, Inc.) and allowed to breathe freely, until ambient CO_2 _levels measured 5%, roughly approximating normal end-tidal CO_2 _(5–5.5%), at which point NO and CO concentrations were measured (Figure 1A). The time taken to reach this CO_2 _concentration was calibrated as a function of animal mass, serving as an index of basal metabolic rate. Typically, the range of time was 6–13 min. for mice weighing 18–35 g, yielding reproducible E_NO _and E_CO _to within 7% across measurements. All measurements were made at times when ambient air levels were typically less than 2 ppb for NO and less than 0.5 ppm for CO (Figure 1B). Air in the chamber was sampled through a stopcock for 20 seconds, at a withdrawal rate of 250 ml/min, such that approximately 83 ml was withdrawn, sufficient to make the needed measurements within a gas sample that constituted less than 15% of the total gas volume within the chamber. The withdrawal rate was set by the gas analyzer flow rate, which was always 250 ml/min for every test. Afterwards, the mice were removed immediately from the chamber and replaced within their cages.

### Airway Responses

Alterations in the enhanced pause (Penh) associated with aerosolized methacholine (MCh) administration was utilized as a measure of AR, as described previously [1]. Briefly, unanesthetized mice were placed within specially designed small volume (~600 ml) Plexiglas chambers equipped with transducers that monitored chamber pressure alterations as a function of mouse breathing patterns (Buxco, Inc.). Penh, a calculated parameter which is a function of the degree of bronchoconstriction in response to MCh, was calculated in the conventional way by a software program (BioSystem XA, Buxco, Inc.) from amplified expiratory pressure signals generated by the transducers [23]. MCh dissolved in phosphate-buffered saline (pH = 7.4) was administered as an aerosol to the mice within the chambers, by using a DeVilbiss ultrasonic nebulizer (aerosol droplet size = 1–5 μm), connected to an aerosol driver and pump apparatus (Buxco, Inc.). The administration duration of each MCh concentration was 2 min., followed by a 3 min. observation and continued data collection period. The highest Penh value achieved during the administration and observation periods was used as the peak response value for each mouse.

### Induction of Airway Inflammation

Mice were sensitized and aerosol challenged with ovalbumin (OVA), using a protocol that reliably increases total cell numbers and eosinophils in recovered bronchoalveolar lavage (BAL) fluid, similar to that previously described [24]. Briefly, sensitization was achieved by using 0.5-ml injections, i.p., of OVA (50 μg/ml, Sigma, Grade VI) and alum (1 mg/ml) as a mild adjuvant, dissolved in normal saline. A mild airway inflammation was induced in which mice were injected twice, once on day one, and then with a booster seven days later. On day 14, the mice were subjected to a 10-minute airway allergen challenge using OVA/saline aerosol (5 mg/ml), which was repeated three times, during days 14 (once, 30 min.) and 15 (twice, 30 min. ea.; 4–6 hours between challenges), with subsequent assessment on day 17.

### Inhibition of NOS and HO

Neuronal/constitutive NOS (NOS-1) was inhibited in NOS-2^-/- ^mice by administration of S-methyl-L-thiocitrulline (SMTC; Alexis) injected i.p. (10 mg/kg) every other day, over a five-day period, which included the week prior to, and the week of, the OVA/saline aerosol challenges. HO was inhibited using tin protoporphyrin (SnPP; Porphyrin Products), dissolved in either DMSO, or aqueous 1 N potassium hydroxide and pH-balanced to a value of *7.0*, injected i.p. (50 μmol/kg), one day prior to aerosol challenge.

### Bronchoalveolar Lavage (BAL)

BAL was performed to verify the induction of airway inflammation by the allergen provocation protocol. We have found that BAL fluid returns can be diminished by 30–50% due to residual MCh-induced bronchoconstriction with the assessment of AR (data not shown), therefore these assessments were performed in a separate group of mice. As previously described [24], forty-eight hours after the last aerosol challenge, the mice undergoing BAL were anesthetized with Metofane, rapidly killed by cervical dislocation, and subjected to open-chest BAL using sterile, endotoxin-free normal saline, to obtain airway cells from both lungs. Differential cell counts were obtained using Diff-Quik staining (Scientific Prod.), and another aliquot was used for cell viability, using the Trypan Blue exclusion method. Two- to three-hundred cells were counted per slide to with total cell counts expressed per body weight of mouse, to normalize for size differences between mice; eosinophils and macrophage numbers were expressed as percent of total cells counted.

### Statistics

Statistical analyses of BAL variables were performed using independent sample t-tests, comparing naïve and OVA treatments within and across mouse strains. E_NO _and E_CO _values without and with airway inflammation within and across mouse strains were compared using independent sample t-tests, while changes in E_NO _over time with induction of AI, and comparisons of AR were made with a repeated-measures ANOVA, with post-hoc testing of discreet data at specific MCh concentrations or times by Student-Neuman Keuls analyses. In cases where the data were assessed to be non-normally distributed, a non-parametric analog (repeated measures ANOVA on ranks) was utilized, followed by a Dunnett's post-hoc test to evaluate differences in discreet data within experimental groups. Changes in E_NO _and E_CO _with inhibition of NOS and HO were made with a paired t-test. Changes in E_NO _with age in IL-10^-/- ^mice were evaluated using an iterative least-squares non-linear regression analysis, modeled with a 3-parameter single exponential decay curve (*y *= *a*·e^-*bx *^+ c, where *y *was the regression-predicted E_NO _at any age point (*x*) on the curve, *a *and *b *were regression constants, *k *was the inverse time constant of decay, i.e., 1/τ, and c was the asymptote of decay). Changes in E_NO _with age in C57Bl6 mice were evaluated using linear regression (*y *= *mx *+ *b*, where *m *was the slope, and *b *was the y-intercept at *x *= 0). The same linear regression approach was used to evaluate E_CO _with age in both IL-10^-/- ^and C57Bl6 mice. All statistical tests and regression analyses were performed using SigmaStat (SPSS, Inc.), in which a value of *P *< 0.05 was considered significant.

## Results

### E_NO _and E_CO _in naïve mice

**Figure 2:**
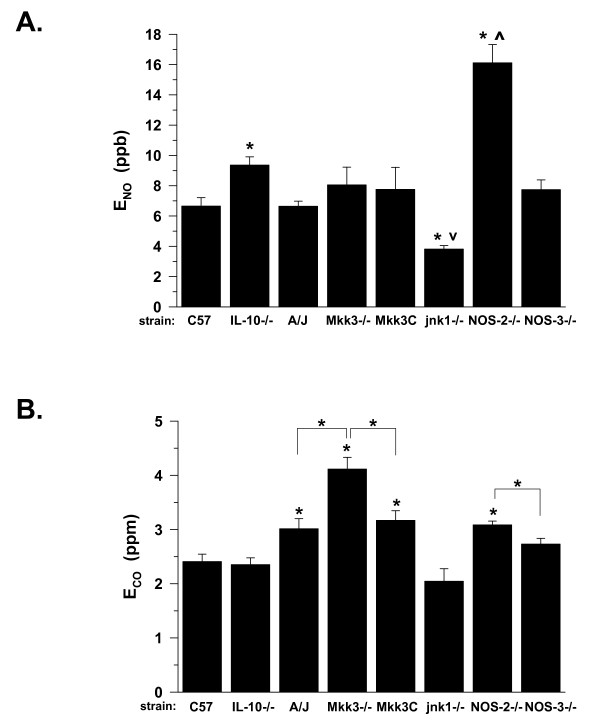
**A**. Exhaled NO (E_NO_) in naïve mice of various strains at 8–22 weeks of age. Values are means ± SE; *P < 0.05 vs. C57Bl6; ^v^*P *< 0.05 JNK1^-/- ^vs. MKK3^-/-^; ^*P *<0.05 NOS-2^-/- ^vs. NOS-3^-/-^; strain(n) = C57Bl6(19), IL-10^-/-^(32), A/J(32), MKK3^-/-^(17), MKK3C (C57/C129(10)), JNK1^-/-^(11), NOS-2^-/-^(14), NOS-3^-/-^(19) **B**. Exhaled CO (E_CO_) in naïve mice. Values are means ± SE, with strain(n), and significance as in panel A; other significant differences as shown by *P<0.05 with brackets.

Average levels of E_NO _in naïve C57Bl6 mice from 8–22 weeks of age ranged from 5–7 ppb (Figure 2A). E_NO _levels in naïve A/J, MKK3^-/-^, MKK3-sufficient (C57/C129) and NOS-3^-/- ^mice were not statistically different from the C57BL6 mice, ranging from 6–9 ppb. However, E_NO _was significantly elevated for IL-10^-/- ^mice, and unexpectedly, for NOS-2^-/- ^mice, to levels of 8–10 and 15–17 ppb (*P *< 0.05), respectively. Notably, E_NO _levels were significantly decreased in JNK1^-/- ^mice. These data suggested that non-invasively measured E_NO _was mouse strain and genetic-knockout dependent, prior to any experimental manipulations.

Average levels of E_CO _in naïve C57Bl6 mice ranged from 2–2.5 ppm (Figure 2B). E_CO _levels in naïve IL-10^-/-^, JNK1^-/-^, and NOS-3^-/- ^mice were not statistically different from the C57Bl6 mice, ranging from 1.5–2.5 ppm. However, E_CO _was significantly elevated in the A/J (~3.0 ± 0.2 ppm, *P *< 0.05), MKK3^-/- ^(~4.0 ± 0.2 ppm, *P *< 0.05), MKK3-sufficient (~3.2 ± 0.2 ppm, *P *< 0.05), and NOS-2^-/- ^(~3.1 ± 0.1 ppm, *P *< 0.05) mice. Furthermore, E_CO _was significantly increased in the MKK3^-/- ^mice as compared to wild-type littermate controls (~4.1 ± 0.2 vs. 3.2 ± 0.2 ppm, *P *< 0.05), and likewise was significantly increased in the NOS-2^-/-^, as compared to NOS-3^-/-^, mice (3.4 ± 0.1 vs. 2.8 ± 0.1 ppm, *P *< 0.05). Similar to E_NO_, these data suggested that non-invasively measured E_CO _was mouse strain and genetic-knockout dependent, prior to any experimental manipulations.

### AR in naïve mice

**Figure 3:**
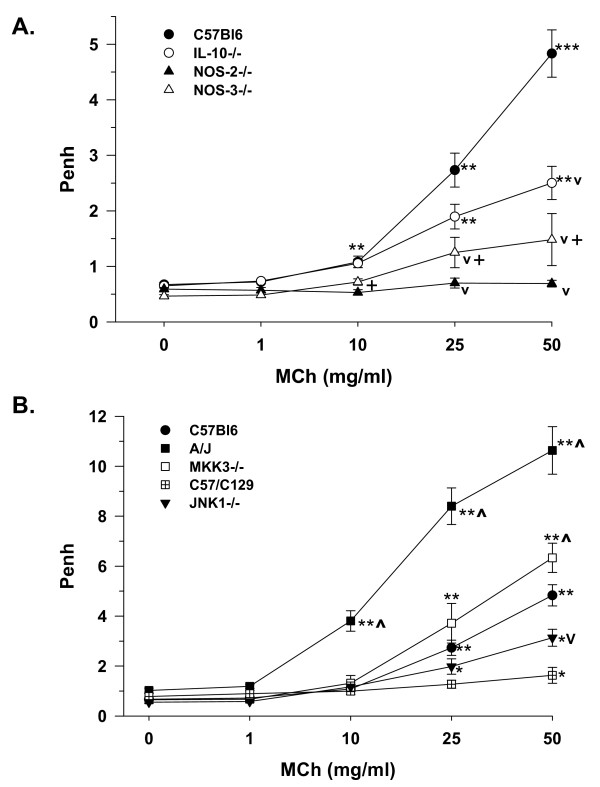
Airway responses to MCh (as enhanced pause; Penh) in naïve mice of various strains. **A**. Means ± SE shown for strains C57Bl6 (filled circle, n = 12), IL-10^-/- ^(open circle, n = 12), NOS-2^-/- ^(filled triangle, n = 8) and NOS-3^-/- ^(open triangle, n = 4); **P < 0.05 vs. 0 mg/ml MCh and next lowest MCh concentration within strain; ***P < 0.05 vs. 0 mg/ml MCh and next two lowest MCh concentrations within strain; ^v^*P *< 0.05 decreased vs. C57Bl6 at same MCh concentration; +*P *< 0.05 NOS-2^-/- ^vs. NOS-3^-/- ^at given MCh concentration. **B**. Means ± SE shown for strains: A/J (filled square, n = 12), MKK3^-/- ^(open square, n = 10), MKK3-sufficient C57/C129 (crossed square, n = 10), JNK1^-/- ^(inverted filled triangle, n = 10), as compared to C57Bl6, as in panel A. *P < 0.05 as compared to 0 mg/ml MCh within strain; ^*P *<0.05 increased vs. C57Bl6 at same MCh concentration; other significance symbols as in panel A.

AR to inhaled MCh was demonstrated in naïve C57Bl6 control mice, such that Penh was significantly increased at all concentrations ≥ 10 mg/ml (Figure 3A). Likewise, naïve IL-10^-/- ^mice demonstrated significant AR, which was of decreased magnitude as compared to the IL-10-sufficient wild-type C57Bl6 mice. NOS-3^-/- ^mice demonstrated a trend toward increased AR at these same MCh concentrations, which was not significantly different from respective *0 *MCh, and was significantly lower than C57Bl6 controls at MCh concentrations above 10 mg/ml (*P *< 0.05). NOS-2^-/- ^mice displayed a nearly flat AR relationship, which was likewise significantly lower (as indicated by inverted carat) than control C57Bl6 mice at MCh concentrations above 10 mg/ml (*P *< 0.05), and was significantly lower than the NOS-3^-/- ^mice at MCh concentrations ≥ 10 mg/ml (*P *< 0.05).

Shown on a greater scale, with C57Bl6 mice for reference, naïve A/J mice demonstrated significant AR to MCh at all concentrations above 1 mg/ml, and this response was significantly greater than that observed in C57Bl6 mice, at these same concentrations (Figure 3B). Interestingly, MKK3^-/- ^mice likewise displayed significant AR at MCh concentrations ≥ 10 mg/ml, and were found to be increased at the highest MCh concentration (50 mg/ml; *P *< 0.05) as compared to C57Bl6 controls. JNK1^-/- ^mice also showed significant AR at 25 and 50 mg/ml, and were significantly less than C57Bl6 mice at the highest concentration of MCh (50 mg/ml, *P *< 0.05). The MKK3-sufficient littermates (C57/C129 mice) were relatively flat in MCh response, demonstrating a small but significant increase only at the highest MCh concentration. These non-invasively obtained AR are consistent with potential mechanisms dependent on airway E_NO _and E_CO _levels, indicated by our exhaled gas measurements.

### E_NO _and E_CO _as a function of age

**Figure 4:**
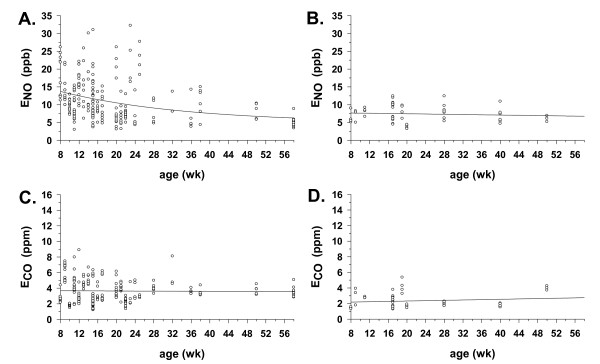
E_NO _and E_CO _as a function of age. **A**. E_NO _with age in IL-10^-/- ^mice (n = 234) from 8–58 weeks of age. Line indicates best-fit exponential decay regression curve (*P *< 0.05), calculated as described in the *Methods *section. Calculated regression equation was: *y *= 5 + 12 e^-0.04*x*^, with r^2 ^= 0.11, and y-intercept (E_NO_) = 13.8 ppb at 8 weeks of age. **B**. E_NO _with age in C57Bl6 mice (n = 51) from 8–50 weeks of age, with abscissa lengthened to 58 weeks for comparison with IL-10^-/- ^mice in panel A. Line indicates best-fit linear regression curve, calculated as described in the *Methods *section. Calculated regression equation was: *y *= - 0.02*x *+ 7.8, with r^2 ^= 0.01, and y-intercept (E_NO_) = 7.7 ppb at 8 weeks of age. **C**. E_CO _with age in same IL-10^-/- ^mice as in panel A. Calculated regression equation was: *y *= - 0.002*x *+ 3.7, with r^2 ^= 0.0002, and y-intercept (E_CO_) = 3.7 ppb at 8 weeks of age. **D**. E_CO _with age in same C57Bl6 mice as in panel B. Calculated regression equation was: *y *= 0.0112*x *+ 2.1, with r^2 ^= 0.02, and y-intercept (E_CO_) = 2.2 ppb at 8 weeks of age.

E_NO _was measured in naive IL-10^-/- ^mice (n = 234) from ages 8 to 58 weeks (Figure 4A). The non-linear regression demonstrated a significant curvilinear relationship of E_NO _with age, beginning at a high point of 13.8 ppb (the *y*-intercept at 8 weeks of age) and declining to an asymptote of 5 ppb, by 58 weeks of age. Unlike the IL-10^-/- ^mice, the naïve C57Bl6 mice tested (n = 51) had a lower *y*-intercept (7.7. ppb at 8 weeks of age) and demonstrated no significant change in E_NO _from ages 8–50 weeks of age, as indicated by a calculated slope value that was near zero over the age range studied (Figure 4B). These results suggest that non-invasively measured E_NO _is stable with age in naïve C57Bl6 mice, but is high and falls with age in the absence of IL-10 and experimental manipulations.

Unlike E_NO _in the IL-10^-/- ^mice, E_CO _was relatively constant with age in the naïve IL-10^-/- ^and C57Bl6 mice tested (Figures 4C and 4D). The y-intercepts at 8 weeks were significantly different from *0 *ppm E_CO _in each case (3.7 and 2.1, respectively), but the slopes of the regression lines were not significantly different from zero, suggesting no significant change over the aging interval monitored.

### E_NO _and E_CO _with induction of allergic airway inflammation

**Figure 5:**
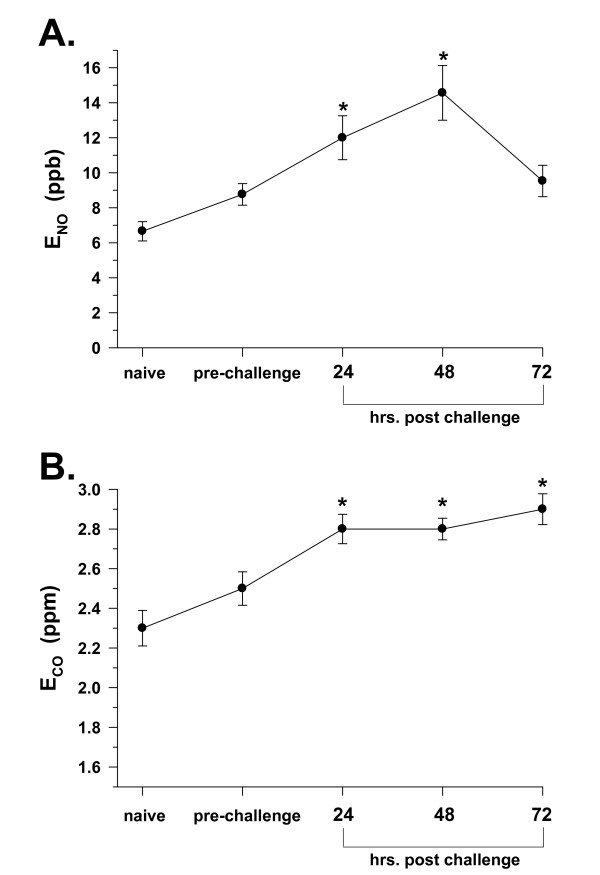
Alterations in E_NO _and E_CO _with induction of inflammation. **A**. Changes in E_NO _with induction of airway inflammation by OVA treatment in C57Bl6 mice, over duration of sensitization and challenge protocol (3 wk.). Naïve is prior to systemic OVA sensitization injection, pre-challenge is after systemic OVA sensitization booster injection, and post-challenge times are elapsed times after 3^rd ^OVA aerosol challenge. Values are means ± SE for n = 20 mice followed over time, *P < 0.05 as compared to naïve. **B**. Changes in E_CO _in same mice as in panel A; *P < 0.05 as compared to naïve.

Allergic airway inflammation, as reflected by BAL total cell count, and macrophage and eosinophil numbers, was produced to varying degrees in all mice with systemic sensitization and airway challenge using OVA (Table 1); however, the total cell count and eosinophil responses were minimal in NOS-2^-/- ^mice. With allergic airway inflammation, a group of C57Bl6 mice (n = 20) followed over time, from the naïve state, through systemic OVA sensitization injection, and subsequent OVA aerosol airway challenge, demonstrated a significant rise in non-invasively measured E_NO _over the course of the inflammation induction protocol. Figure 5A indicates an average starting point of nearly 7 ppb, with a significant doubling to approximately 12 (*P *< 0.05) and 14 (*P *< 0.05) ppb, at 24 and 48 hr after the final OVA aerosol challenge, followed by a decline to approximately 10 ppb at 72 hr (n.s.). This pattern was suggestive of an early to intermediate response of E_NO _with induction of allergic airway inflammation, followed by a reduction in the late-phase portion of the inflammatory response.

**Table 1:** Bronchoalveolar lavage cell counts in various strains of mice.

Strain	Treatment	Total cells (× 10^4 ^/g)	Macrophages (%)	Eosinophils (%)
C57Bl6	-OVA	1.4 ± 0.1	99 ± 1	0
	+OVA	8.5 ± 1.6*	51 ± 14*	48 ± 14*
IL-10^-/-^	-OVA	0.5 ± 0.1	99 ± 1	0
	+OVA	4.5 ± 1.0*	39 ± 5*	45 ± 8*
A/J	-OVA	0.5 ± 0.1	96 ± 1	0
	+OVA	5.2 ± 0.5*	16 ± 3*	79 ± 4*
MKK3^-/-^	-OVA	0.5 ± 0.1	91 ± 4	0
	+OVA	2.3 ± 0.5*	66 ± 7*	44 ± 5*
C57/C129	-OVA	0.8 ± 0.1	93 ± 2	0
	+OVA	2.1 ± 0.3*	96 ± 2	1 ± 1
JNK1^-/-^	-OVA	1.2 ± 0.4	93 ± 3	1 ± 1
	+OVA	0.9 ± 0.3	24 ± 8*	20 ± 8*
NOS-2^-/-^	-OVA	0.2 ± 0.1	38 ± 17	0
	+OVA	0.3 ± 0.1	75 ± 14*	1 ± 1

-OVA = naïve; +OVA = airway inflammation; total cell counts expressed per gram body weight; values are means ± SE; *P < 0.05 +OVA vs. -OVA for each respective strain; n = 3–7 per group.

Similarly, in those same mice, E_CO _was increased with induction of allergic airway inflammation, rising from 2.3 ppm in the naïve state, to 2.8 ppm over the 24–72 hr time interval after the final OVA aerosol challenge (Figure 5B). This pattern was consistent with an early response of E_CO _with induction of allergic airway inflammation, but lacked the fall at 72 hr observed with E_NO_, suggesting a lack of resolution within the same time interval as that observed for E_NO_.

### E_NO _and E_CO _comparisons with allergic airway inflammation

**Figure 6:**
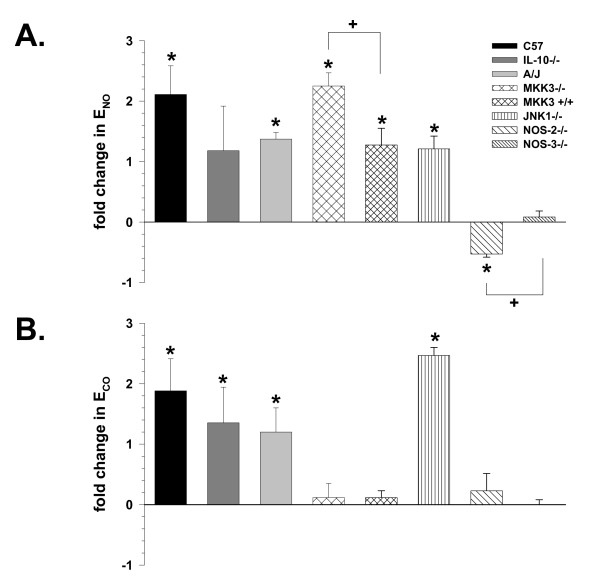
Comparisons of E_NO _and E_CO _across mouse strains. **A**. Exhaled NO (E_NO_) with airway inflammation, expressed as fold change as compared to E_NO _in respective-strain naïve mice. *P < 0.05 vs. respective-strain naïve mice controls; +*P *< 0.05 MKK3^-/- ^vs. MKK3-sufficient (C57/C129) controls, and *P *< 0.05 NOS-2^-/- ^vs. NOS-3^-/- ^mice. Values are means ± SE; strain(n) = C57Bl6(8), IL-10^-/-^(12), A/J(12), MKK3^-/-^(11), C57/C129(6), JNK1^-/-^(4), NOS-2^-/-^(8), NOS-3^-/-^(14). **B**. Changes in exhaled CO (E_CO_) with induction of airway inflammation, expressed as fold change as compared to E_CO _in respective-strain mice. Values are means ± SE, with significance and n as in Panel A.

With allergic airway inflammation, the levels of E_NO _significantly increased from 1–3-fold (*P *< 0.05) in C57Bl6, A/J, MKK3^-/-^, MKK3-sufficient, and JNK1^-/- ^mice (Figure 6A), when compared to E_NO _values from each respective naïve strain or knockout. The numerical increase in E_NO _in IL-10^-/- ^mice suggested a similar increase, but was not statistically different from the naïve IL-10^-/- ^E_NO _values. E_NO _of the MKK3^-/- ^mice with allergic airway inflammation was greater (*P *< 0.05) than its MKK3-sufficient littermate control. However, as compared to both C57Bl6 and NOS-3^-/- ^mice, the NOS-2^-/- ^mice demonstrated a significant reduction in E_NO _with allergic airway inflammation (-50%, *P *< 0.05), while E_NO _of the NOS-3^-/- ^mice remained unchanged from baseline levels and was different than the NOS-2^-/- ^mice. These data suggested that different responses to allergic airway inflammation were dependent on the strain or targeted gene knockout of the mice tested, as measured with our non-invasive technique.

E_CO _was significantly increased ~1–2.5-fold in C57Bl6, IL-10^-/-^, A/J, and JNK1^-/- ^mice with airway inflammation (Figure 6B), as compared to respective naïve strain or knockout mice. No significant change in E_CO _was observed in MKK3^-/-^, MKK3-sufficient, NOS-2^-/-^, and NOS-3^-/- ^mice, compared to their appropriate littermate controls. These data suggested different responses of E_CO _as compared to E_NO_, as a function of allergic airway inflammation and mouse strain or targeted gene knockout.

### Effects of NOS and HO inhibition

**Figure 7:**
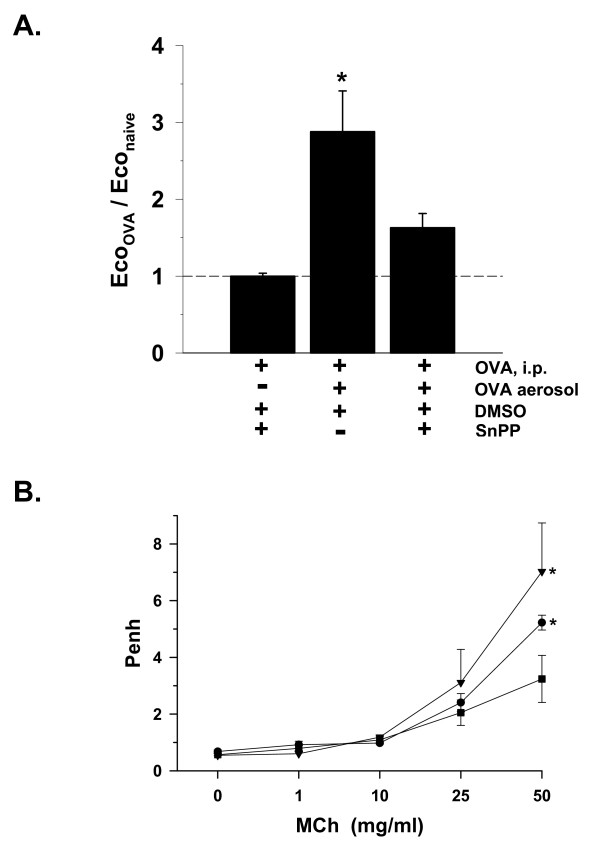
Changes in E_CO _and AR with inhibition of HO. **A**. Exhaled CO (E_CO_) changes with inhibition of heme-oxygenase by tin protoporphyrin (SnPP) without induction of airway inflammation (OVA, i.p.) and with airway inflammation (+OVA aerosol), in C57Bl6 mice, expressed as fraction of naïve (-OVA) controls (dashed line average = 2.3 ppm E_CO_, as shown in Figure 2A). *P < 0.05 vs. both naïve baseline and respective strain OVA sensitized-only (i.p.) group; n = 4/bar. **B**. Airway responses (Penh) to methacholine (MCh) aerosol in C57Bl6 mice either naïve (filled square ; n = 11); with airway inflammation (+OVA) +DMSO vehicle (filled circle; n = 6); or with airway inflammation + SnPP/DMSO (inverted filled triangle; n = 4); *P < 0.05 increased vs. naïve at same MCh concentration.

As noted previously in Figure 5B, systemic sensitization with i.p. injections of OVA in untreated C57Bl6 control mice did not significantly increase E_CO_, but challenge with OVA aerosol resulted in an increase in E_CO_, indicating that the rise in E_CO _was associated with induction of allergic airway inflammation (Figure 7A). This increase in E_CO _was attenuated in mice pretreated with SnPP to inhibit HO. The degree of attenuation made it statistically similar to both the non-SnPP-treated group with airway inflammation, and the non-SnPP-treated naïve controls, which suggested that the value was intermediate between them.

As compared to naïve C57Bl6 mice, AR increased at the highest concentration of MCh (50 mg/ml, *P *< 0.05) in C57Bl6 mice with allergic airway inflammation due to OVA (Figure 7B). With SnPP administration in the presence of allergic airway inflammation, AR was also elevated at the highest concentration of MCh as compared to naïve controls (*P *< 0.05), but was not statistically different from that measured in mice treated with OVA and no SnPP. These data were suggestive of a trend toward increased AR with inhibition of HO in the presence of allergic airway inflammation.

**Figure 8:**
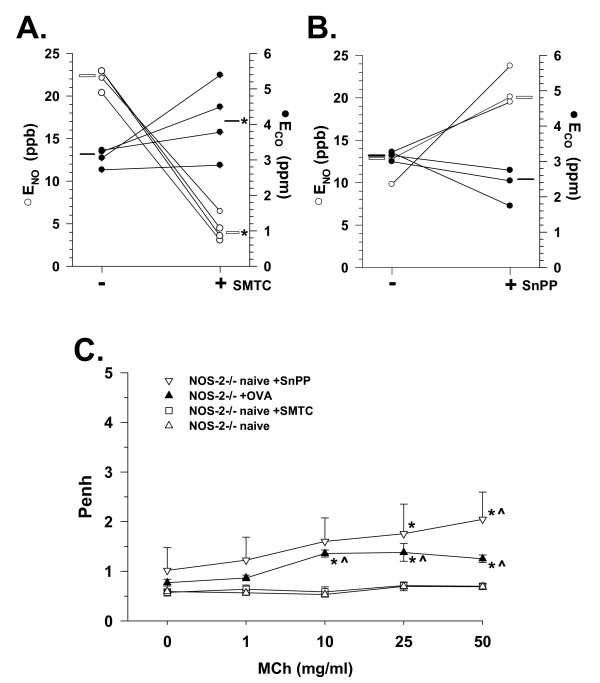
Modulation of E_NO_, E_CO_, and AR in NOS-2-/- mice. **A**. Changes in exhaled NO (E_NO_; open circle) and CO (E_CO_; filled circle) in NOS-2^-/- ^mice (n = 4) 48 hr. after administration of S-methyl-L-thiocitrulline (SMTC). Open bar indicates median E_NO _value, black bar indicates median E_CO _value; *P < 0.05 vs. -SMTC. **B**. Changes in exhaled NO (E_NO_; open circle) and CO (E_CO_; filled circle) in NOS-2^-/- ^mice (n = 3) 48 hr. after administration of tin protoporphyrin (SnPP). Symbols and bars as in panel A. **C**. Airway responses (Penh) to methacholine (MCh) aerosol in naïve NOS-2^-/- ^mice treated with SMTC (open square n = 4) or SnPP (inverted open triangle; n = 3), and with allergic airway inflammation (+OVA, filled triangle, n = 8) as compared to naive NOS-2^-/- ^mice with no treatment (open triangle; n = 8). *P < 0.05 vs. 0 mg/ml MCh, ^*P *< 0.05 vs. naive at same MCh concentration.

In naive NOS-2^-/- ^mice, a significant reduction in E_NO _from a median value of 22.5 ppb to 4 ppb (*P *< 0.05) was observed after chemical inhibition of neuronal/constitutive NOS (I) with SMTC (Figure 8A). These declines in E_NO _with SMTC administration were associated with a significant rise in E_CO _from a median value of 3.1 to 4.1 ppm (*P *< 0.05). Conversely, administration of SnPP to naïve NOS-2^-/- ^mice resulted in a decline in E_CO _in each of the three mice tested, along with concomitant increases in E_NO _in those same mice (Figure 8B). Statistical analyses were not applied to those data due to the small number of mice tested, however, the respective E_CO _and E_NO _responses were similar in direction and magnitude. These data indicated that in the absence of NOS-2, inhibition of NO production by NOS-I can result in increases in CO levels, and that inhibition of CO production by HO may result in increased NO levels.

AR was increased in NOS-2^-/- ^mice with induction of allergic airway inflammation by OVA (Figure 8C), which was consistent with the reduction of E_NO _observed in this group. AR was also increased in naive NOS-2^-/- ^mice with inhibition of HO by SnPP. However, inhibition of NOS-I with SMTC did not alter AR to MCh as compared to naive NOS-2^-/- ^mice, remaining flat across MCh concentrations. These data suggested that NO-inhibition driven changes in E_CO _may be associated with alterations in AR to MCh.

## Discussion

Our results indicate that a simple non-invasive technique utilized for measurement of E_NO _and E_CO _can be associated with physiological responses in specific strains of mice, and with specific manipulation of important inducible enzymes, like NOS and HO. This technique also allowed collection of repeated measurements over time, making it useful in determining alterations in E_NO _and E_CO _with induction of acute experimental airway inflammation.

### Alterations in E_NO_

The levels of non-invasively measured E_NO _in naïve C57Bl6 and A/J mice are consistent with prior findings in mice using similar, but more complicated measurement techniques using flow-through null-gas or gas scrubbers [17,18]. The exceptions to those levels were our findings in IL-10^-/-^, NOS-2^-/-^, and JNK1^-/- ^mice. A high level of E_NO _would be expected in IL-10^-/- ^mice [24], because IL-10 is a natural suppressor of NOS-2 induction, and consequent NO production [25]. However, the elevated level of E_NO _in the NOS-2^-/- ^mice was an unexpected finding, based on one prior report [26], and given that the major inducible enzyme producing NO was missing. Prior reports have alternatively suggested that a significant amount of NO in the murine airway is due to the action of NOS-3 [27], or NOS-1 [26]. Selective inhibition of NOS-1 in the NOS-2^-/- ^mice with SMTC resulted in a decrease in E_NO _toward levels seen in the other strains, which would lead us to conclude that upregulation of NOS-1 was a likely source of the increased E_NO _in the NOS-2^-/- ^mice.

Repeated non-invasive measures of E_NO _in C57Bl6 mice from their naïve state to induction of airway inflammation indicated a pattern of increase consistent with an early inflammatory response that resolved over time. While not unexpected, those data demonstrate that our simple method can follow E_NO _as a biomarker of the allergic airway inflammatory response in wild-type C57Bl6 mice, similar to humans [28]. An unexpected finding was that administration of OVA resulted in decreased E_NO _in NOS-2^-/- ^mice, previously measured to be high when naive (Figures 2 and 6), coupled with the lack of cell recruitment and eosinophilia (Table 1). There is some precedent for these responses, reported as decreased nitrite and nitrate [29], and a lack of eosinophilia in BAL fluid of NOS-2^-/- ^mice with allergic airway inflammation, as compared to C57Bl6 mice [27,30]. However, that precedent has not been uniform, in that at least one report indicated no differences in allergic airway eosinophilia between C57Bl6 and NOS-2^-/- ^mice [31]. Furthermore, NOS-3 overexpression in mice has been associated with decreased allergic inflammation-associated airway eosinophilia [29]; therefore, its compensatory upregulation in NOS-2^-/- ^mice could explain our findings.

### Associations of E_NO _with AR

In several instances, e.g., C57Bl6 and IL-10^-/- ^mice, the respective measured low and high E_NO _levels were consistent with the observed AR to MCh, supporting the idea that NO may act as a bronchodilator [8]. The high level of E_NO _in the NOS-2^-/- ^mice was unexpected, but again, a concomitant low level of AR was consistent with NO counteracting the effects of MCh on AR. Those findings were consistent with prior findings in NOS-1^-/- ^mice [31], supporting that the absence or inhibition of one or more NOS isoforms can influence non-invasively measured AR to MCh. Furthermore, the unexpected reduction in E_NO _with allergic airway inflammation in the NOS-2^-/- ^mice appears to fit with the increase in AR we observed (Figure 8C), and is consistent with elevations reported in NOS-2^-/- ^mice given OVA, using a more invasive bronchospasm assessment method [27,30]. However, it also has been reported that NOS-3 may be a significant factor determining AR in mice [27]. Although reasonably selective for NOS-1, SMTC is also known to inhibit NOS-3 [32]. Therefore, we cannot rule out the possibility that the effects of SMTC were not solely on NOS-1, but also may have involved inhibition of NOS-3, resulting in the AR responses we observed. The increased AR demonstrated by the naive NOS-3^-/- ^mice in comparison with the naive NOS-2^-/- ^mice (Figure 3) would suggest that compensatory NOS-3 upregulation was responsible for the relatively flat AR relationship in the NOS-2^-/- ^mice. Furthermore, the lack of effect of SMTC on AR in the face of significantly reduced E_NO _levels in the NOS-2^-/- ^mice (Figure 8) suggests that NOS-1 was not acting to suppress AR with genetic deletion of NOS-2.

### E_NO _with Aging in the Absence of IL-10

In our prior studies of IL-10^-/- ^mice demonstrating elevated E_NO _values, the mice that were observed with symptoms of rectal prolapse, diarrhea, and failure to gain weight, were routinely excluded from consideration [24,33,34], due to the tendency of IL-10^-/- ^mice to develop enterocolitis with age [20]. However, throughout those studies and the present one, we noted either a lack of, or long-delayed (>22 weeks of age), onset of those outward symptoms in some groups of IL-10^-/- ^mice, allowing them to live beyond their normal life expectancy, making us curious about their E_NO _levels as they aged. This provided an opportunity for us to determine the relationship of age and E_NO_, in a mammalian system lacking IL-10, in comparison with their wild-type IL-10 sufficient C57Bl6 counterparts. Our findings suggest that the initially high E_NO _associated with a lack of IL-10 declines with age.

While the typical inflammation-associated symptoms were either absent or marginally discernable in aging IL-10^-/- ^mice in the present studies, it could be argued that there were either undetermined levels of internal inflammation not outwardly apparent, or that those mice were somehow protected from the effects of diffuse inflammation, and specifically those occurring within the gastro-intestinal tract, that allowed their continuation as survivors. Our experiments thus far do not allow us to discern which of these possibilities occurred. However, prior studies suggest the potential for compensatory mechanisms [27,31,35,36] that may have resulted in less inflammation and therefore, decreased NO production, which would be consistent with our findings.

### Alterations of E_CO_

E_CO _was similar in C57Bl6, IL-10^-/-^, JNK1^-/-^, and NOS-3^-/- ^mice, being most strongly elevated in naïve MKK3^-/- ^mice, perhaps as a result of disruption of the normal MAPK pathway through which CO is known to signal [37]. Therefore, while E_CO _levels appear to be associated with airway inflammation and asthma severity [16], our data support the idea that a portion of this association may be through the involvement of MAPK pathways modulated as part of the process of inflammation. Elevated levels of E_CO _observed in naïve A/J and NOS-2^-/- ^mice are difficult to interpret within this MAPK-associated construct, but suggest other mechanisms modulating E_CO _that require investigation within those mouse strains. Following development of airway inflammation, several strains of mice (MKK3^-/-^, NOS-2^-/-^, and NOS-3^-/-^) had no measurable changes in E_CO_, in contrast to many others. In the case of the MAPK, those data would suggest that MKK3 is a critical MAPK that promotes an airway CO response to airway inflammation, whereas JNK1 is not. Furthermore, our data suggest that, similar to E_NO_, E_CO _also tracks development of acute allergic airway inflammation in C57Bl6 mice, as has been reported in humans [11], making Eco a potentially useful biomarker in mouse models of asthma characteristics.

### Associations of E_CO _with AR

Exogenously administered CO has been shown to function as a bronchodilator and to reduce AR in guinea pigs and mice [1,3], thus these properties might likewise be expected of endogenous CO in the airway. The reduction of E_CO _with SnPP treatment in C57Bl6 mice, coupled with the associated increase in AR (Figure 7), support this concept. An increase in AR was also observed with SnPP treatment and reduction of E_CO _in NOS-2^-/- ^mice, while E_NO _increased (Figure 8). Taken together, these data suggest that airway CO may be produced in high amounts as a bronchoprotective agent in some strains of mice, and that its affects may be dependent on its balance with airway NO production.

### Critique of Methods

One of the primary goals of this study was to make simple non-invasive assessments of airway NO and CO, as E_NO _and E_CO_, and to correlate them with differences in AR, likewise measured non-invasively, as Penh. The advantages of the techniques utilized in the present study were their non-invasive assessment, repeatability, and minimal manipulation of unanesthetized mice. The procedures were well-tolerated and resulted in no discernable immediate or long-lasting effects, either outwardly, or in the variables measured.

The use of Penh as an index of MCh-driven airway constriction, or its associated events, has been questioned recently, due to dissociations in airway resistance and Penh measurements reported under some conditions, in certain strains such as BALB/c and C57Bl6 mice [38]. We have found previously that, in our hands, Penh does correlate with allergic AI-driven changes in airway resistance (as measured by invasive techniques), in strains we have tested, such as C57Bl6 and A/J mice [1,33]. Furthermore, we have observed that Penh measurements of MCh-associated airway responses match MCh-stimulated airway smooth muscle responses of tracheal rings *in vitro*, in C57Bl6 and IL-10^-/- ^mice [33]. Moreover, our results were corroborated by the studies of Justice, et al., [39] on C57Bl6 and IL-10^-/- ^mice, in which the patterns of MCh-associated Penh measurements tracked both airway resistance and tracheal ring tension measurements in those same mice. Finally, our Penh data indicating increments in AR in NOS-2^-/- ^mice given OVA are consistent with a prior report in NOS-2^-/- ^mice using a more invasive approach to measure responses to MCh [27,30]. Although the magnitude of increment reported in that study appeared greater, that magnitude difference could be explained by their strong OVA challenge protocol, which administered OVA aerosol 12 times over 6 days, as compared to our mild challenge protocol. Thus, those prior demonstrations of applicability, coupled with the goals of the present study, which were to determine associations between non-invasively measured variables, can be considered to meet the requirements for the utilization of Penh, as suggested by De Lorme and Moss [40].

A 3-parameter single exponential decay curve was utilized to assess the relationship of E_NO _with age in IL-10^-/- ^mice, whereas a simple linear regression was utilized for the C57Bl6 mice, based on the visual appearance of the data in each case. For completeness, a simple linear regression was also performed on data from the IL-10^-/- ^mice, which yielded a similar y-intercept at 8 weeks of age (E_NO _= 14.1 ppb), and a similar regression correlation coefficient (R^2 ^= 0.11), as compared to the exponential approach. A shallow, but significant negative slope (-0.2 ppb/week; *P *< 0.05) was obtained with the linear method, consistent with the exponential decay using the other method. It must be noted that although we obtained statistically significant regressions for E_NO _vs. age for mice missing IL-10, the correlation coefficient indicates that age accounts for just over 10% of the decline in E_NO_, i.e, nearly 90% of the decline may be attributable to other factors that may or may not be associated with age, per se. Even so, these data represent the first attempt to characterize E_NO _with age in mice, which was observed to be low and unchanging in wild-type C57Bl6 mice, and initially high and declining, when IL-10 is missing. Further study is necessary to identify the factors behind these relationships.

## Conclusion

Our E_NO _and AR findings are generally consistent with compensatory physiologic alterations occurring in NOS-2^-/- ^mice [29,30], that NOS-1 can contribute significantly to the measured E_NO _level [26], and that NOS-3 may contribute less to E_NO_, but may have an influence on AR [31]. Therefore, our simple, non-invasively-measured E_NO _and E_CO _data indicate that those measures can be used as biomarkers of inflammation kinetics, and potential airway physiologic alterations when critical NO- and CO-producing enzymes are missing or inhibited.

## List of Abbreviations

ANOVA: analysis of variance; AI: airway inflammation; AR: airway responses; BAL: broncheoalveolar lavage; CO: carbon monoxide; DMSO: dimethyl sulfoxide; E_NO: _exhaled nitric oxide; E_CO:_ exhaled carbon monoxide; HO: heme-oxygenase; HO-1: heme-oxygenase-1; IL-10: interleukin-10; MCh: methacholine; JNK-1: c-jun activated kinase 1; MAP:  mitogen-activated protein kinase; MKK3: MAP kinase kinase 3; NO: nitric oxide; NOS: nitric oxide synthase; NOS-1: neuronal nitric oxide synthase; NOS-2: inducible nitric oxide synthase; NOS-3: endothelial nitric oxide synthase; OVA: ovalbumin; Penh: enhanced pause; ppb: parts per billion; ppm: parts per million; SMTC: S-methionyl-L-thiocitrulline; SnPP: tin protoporphyrin; SPF: specific pathogen free

## Competing interests

The authors declare that they have no competing interests.

## Authors' contributions

JMS participated in the development of the non-invasive technique for measuring E_NO _and E_CO_, direction of the gas micro-analyzer utilization and maintenance, analysis of the data, and helped to draft the manuscript, AMKC provided funds for the study, provided the gas micro-analyzer, assisted with data interpretation, and helped to draft the manuscript, WJC provided funds for the study, assisted with data interpretation, and helped to draft the manuscript, BTA provided laboratory facilities and equipment, supervised and directed the study and technical support staff, participated in development of the non-invasive technique for measuring E_NO _and E_CO_, assisted with data interpretation and analyses, and helped to draft the manuscript. All authors read and approved the final manuscript.
